# Supraclavicular thermography does not isolate a brown adipose tissue signal during room temperature β‐adrenergic stimulation in adults

**DOI:** 10.14814/phy2.71202

**Published:** 2026-11-06

**Authors:** Taylor R. Ewell, Christina M. N. Cheng, Matthew C. Bomar, Allison M. Tripure, David M. Brown, Reagan L. Brown, David P. Thomson, Blair D. Johnson, Christopher Bell

**Affiliations:** ^1^ Department of Health and Exercise Science Colorado State University Fort Collins Colorado USA; ^2^ Department of Kinesiology Indiana University‐Bloomington Bloomington Indiana USA

**Keywords:** energy expenditure, indirect calorimetry, isoproterenol, mirabegron, skin temperature, sympathoadrenal

## Abstract

Brown adipose tissue (BAT) is a potential therapeutic target for cardiometabolic diseases and is activated by β‐adrenergic receptor (β‐AR) signaling. Two randomized, single‐blind, placebo‐controlled crossover trials examined responses to non‐selective intravenous β‐AR stimulation in healthy adults, with co‐administration of an oral β3‐AR agonist (mirabegron; Study 1) or an intravenous antioxidant (vitamin C; Study 2). Placebo data were pooled (*n* = 28, 14 M/14F). Thermogenesis was assessed (graded isoproterenol infusion, 6, 12, and 24 ng/kg fat‐free mass/min, 30 min each). Energy expenditure and supraclavicular and reference‐region skin temperatures were measured using indirect calorimetry and infrared thermography (IRT), respectively. Baseline energy expenditure was 1.06 ± 0.26 kcal/min. Isoproterenol increased energy expenditure at each dose (0.09 [0.05, 0.13], 0.13 [0.09, 0.18], and 0.20 [0.13, 0.26] kcal/min; mean differences [95% CIs]; all *p* < 0.001). Supraclavicular skin warming was not regionally specific: although it warmed at the highest dose (0.56°C [0.24, 0.88], *p* < 0.001), the sternum warmed more, exceeding it by 0.70°C [0.28, 1.12] (*p* < 0.001). Mirabegron increased energy expenditure (0.07 [0.04, 0.10] kcal/min, *p* < 0.001) without raising supraclavicular skin temperature (*p* = 0.29); vitamin C did not augment thermogenesis. Isoproterenol increased whole‐body thermogenesis, but supraclavicular skin IRT did not isolate a BAT‐specific thermal signal. Supraclavicular skin warming alone should be interpreted cautiously as evidence of β‐AR‐mediated BAT activation under room‐temperature conditions.

## Graphical abstract

Two randomized, single‐blind, placebo‐controlled crossover trials examined responses to non‐selective intravenous β‐AR stimulation (isoproterenol) in healthy adults, with co‐administration of an oral β3‐AR agonist (mirabegron; Study 1) or an intravenous antioxidant (vitamin C; Study 2). Energy expenditure and supraclavicular and reference‐region skin temperatures were measured using indirect calorimetry and infrared thermography, respectively. Non‐selective intravenous β‐AR stimulation increased energy expenditure and supraclavicular and reference‐region skin temperatures. Co‐administration of an oral β3‐AR agonist augmented the increase in energy expenditure without raising supraclavicular skin temperature. Co‐administration of an intravenous antioxidant did not augment thermogenesis. Non‐selective β‐AR stimulation increased whole‐body thermogenesis, but supraclavicular skin thermography did not isolate a brown adipose tissue (BAT)‐specific thermal signal. Supraclavicular skin warming alone should be interpreted cautiously as evidence of β‐AR‐mediated BAT activation under room‐temperature conditions.

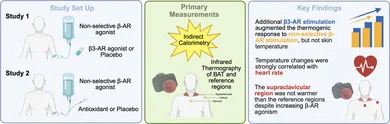

## 1 INTRODUCTION

Brown adipose tissue (BAT) is a metabolically active tissue in adult humans (Saito et al., 2009). BAT has garnered considerable interest as a potential therapeutic target for cardiometabolic diseases, such as obesity and type 2 diabetes (Cheng et al., 2021), due to its thermogenic (Cannon & Nedergaard, 2004) and endocrine (Gavaldà‐Navarro et al., 2022) functions. BAT activation is classically attributed to β‐adrenergic receptor (β‐AR) signaling. In rodents, β3‐AR stimulation is the primary driver of BAT activation (Cannon & Nedergaard, 2004); however, the β‐AR subtype(s) responsible for activation in humans remain less clearly defined as β1 (Riis‐Vestergaard et al., 2020)‐, β2 (Blondin et al., 2020; Straat et al., 2023)‐, and β3 (Cero et al., 2021; Cypess et al., 2015)‐ARs have each been associated with BAT activation. In contrast, each of these β‐AR subtypes has also been shown to be not critical for human BAT thermogenesis (Cero et al., 2021; Füchtbauer et al., 2026; Riis‐Vestergaard et al., 2020), and this controversy remains unresolved (Cypess et al., 2025). Critically, human sympathetic activation of β‐ARs is inherently non‐selective.

Much of the human BAT literature relies on either cold exposure (Iwen et al., 2017; Ouellet et al., 2012; van der Lans et al., 2013) or selective adrenergic agonists (Cypess et al., 2015; Riis‐Vestergaard et al., 2020; Straat et al., 2023). Each of these approaches has important limitations. While cold exposure promotes sympathoadrenal action, it also activates other potentially confounding thermoregulatory mechanisms, including α‐adrenergic vasoconstriction, to defend core temperature. Thus, cold exposure does not isolate the β‐adrenergic component of the response (Charkoudian, 2003). Selective agonists do not reflect endogenous sympathoadrenal activation. Non‐selective β‐AR stimulation, such as intravenous delivery of isoproterenol, more closely mimics endogenous endocrine action, yet relatively few studies have examined BAT responses under non‐selective β‐AR stimulation. Those that have are limited by small sample sizes (typically *n* ≤ 12) and narrow homogeneous populations consisting primarily of lean, healthy males (Carey et al., 2015; Vosselman et al., 2012). This is a critical knowledge gap because women are a historically understudied population in biomedical research (Mazure & Jones, 2015) and recent studies suggest there may be sex differences in β‐AR‐mediated physiology (Jacob et al., 2025). Further, in light of the prevalence of overweight and obesity, lean healthy males are not reflective of the majority of the population in the United States (Overweight & Obesity Statistics, n.d.). BAT abundance and adrenergic responsiveness are influenced by sex (Kaikaew et al., 2021) and adiposity (Kulterer et al., 2022), raising questions regarding the generalizability of existing findings to broader populations.

Beyond cohort breadth, mechanistic interrogation of BAT's contribution to non‐selective β‐AR‐mediated thermogenesis benefits from complementary pharmacological probes. Co‐administering the β3‐AR agonist mirabegron with non‐selective β‐AR stimulation tests whether adding β3‐AR stimulation augments the thermogenic response. A larger response under combined stimulation would be consistent with a β3‐AR‐sensitive contribution to that response; although this approach cannot definitively establish receptor specificity or the contribution of BAT. Additionally, prior work has demonstrated that acute antioxidant administration can augment β‐AR‐mediated physiological function (Mak & Newton, 2001; Shinke et al., 2007), including thermogenesis (Bell et al., 2006), in populations with elevated oxidative stress (Bell et al., 2006), implicating reactive oxygen species as a modulator of β‐AR signaling. This may have important relevance for β‐AR mediated BAT function given the electron transport chain is a major source of reactive oxygen species, and BAT is characterized by high mitochondrial density.

To be able to optimally exploit BAT for therapeutic purposes, a thorough appreciation of the mechanisms of BAT regulation is of clear clinical and biomedical importance. Therefore, the primary purpose of the current work was to characterize thermogenic responses to non‐selective β‐AR stimulation, under room‐temperature conditions, in a cohort of men and women spanning a wider range of body composition than typically studied previously. Secondary aims were to (1) determine whether β3‐AR stimulation with mirabegron augments the thermogenic response to non‐selective β‐AR stimulation, and (2) determine whether acute vitamin C administration modulates the thermogenic response to non‐selective β‐AR stimulation. We hypothesized that (1) non‐selective β‐AR stimulation would elicit dose‐dependent thermogenic responses across the cohort, with the magnitude of increase inversely related to adiposity; (2) mirabegron would augment the thermogenic response to non‐selective β‐AR stimulation, consistent with a β3‐AR‐mediated BAT contribution; and (3) acute vitamin C administration would augment the thermogenic response to non‐selective β‐AR stimulation.

## 2 METHODS

### 2.1 Ethical approval

The protocols were approved by the Colorado State University Institutional Review Board (Study 1: protocol #2981, January 2021; Study 2: protocol #6115, December 2024) and conformed to the standards of the Declaration of Helsinki. All participants provided written informed consent before participation. The trials were registered at ClinicalTrials.gov (NCT06011265 and NCT07341308). Study 1 took place from April to December 2023; Study 2 took place from March to November 2025.

### 2.2 Study designs

In both studies, participants reported to the laboratory in the morning to undergo assessment of resting and β‐AR‐mediated thermogenesis (Figure 1). β‐AR‐mediated thermogenesis was evoked via graded, continuous intravenous infusion of a non‐selective β‐AR agonist, isoproterenol (ISO), comprising three consecutive 30‐min infusions (6, 12, and 24 ng/kg fat‐free mass (FFM)/min). Every experimental visit incorporated the isoproterenol infusion. Prior to isoproterenol infusion, participants received either placebo or a perturbation. In Study 1, the perturbation was ingestion of the β3‐AR agonist, mirabegron (100 mg; Myrbetriq, Astellas Pharma Inc); the placebo was an empty gelatin capsule. In Study 2, the perturbation was co‐infusion of ascorbic acid at a dose previously shown to augment β‐AR‐mediated thermogenesis (Bell et al., 2006) (vitamin C, 0.05 g/kg FFM delivered at a rate of 1 mL/min; ASCOR, McGuff Pharmaceuticals) dissolved in 100 mL saline; the placebo was saline only. This infusion was initiated during the lead‐in phase and continued throughout the isoproterenol delivery.

**FIGURE 1:**
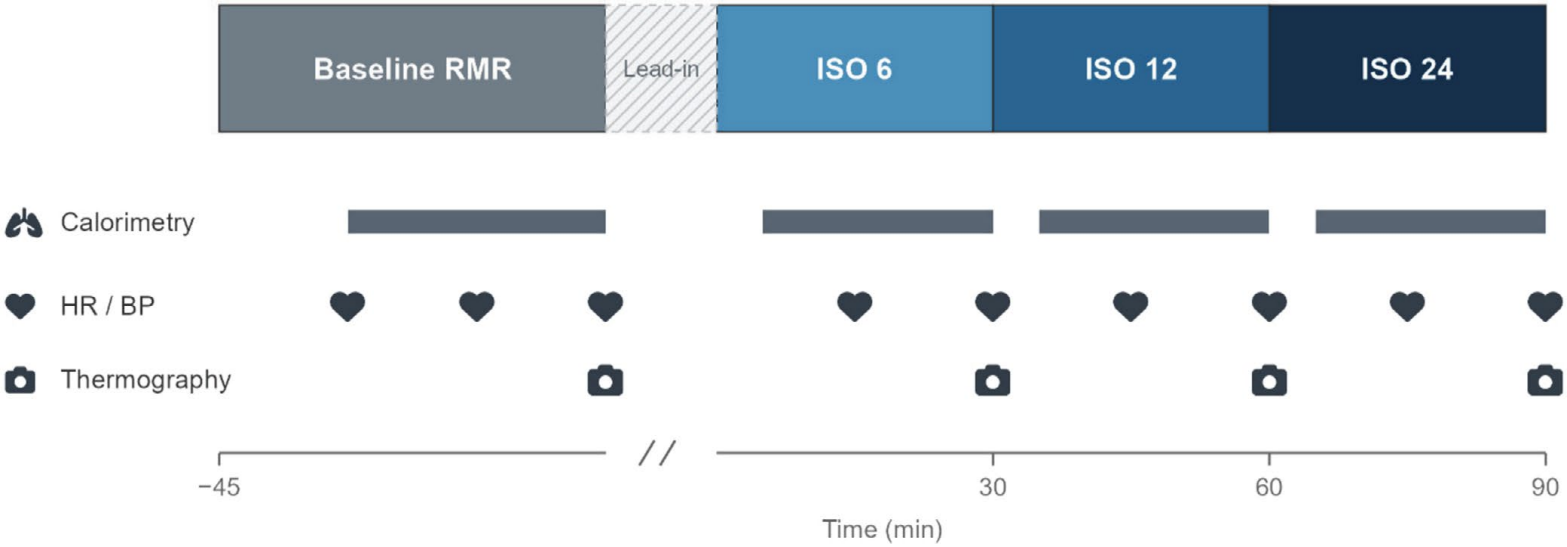
Experimental protocol overview. Participants completed baseline resting metabolic rate assessment, a study‐specific lead‐in period, and three 30‐min isoproterenol infusion blocks at 6, 12, and 24 ng/kg fat‐free mass/min. The lead‐in period was 30 min in Study 1 after mirabegron/placebo ingestion and 15 min in Study 2 during vitamin C/saline infusion initiation. Measurements were collected as shown. BP, blood pressure; FFM, fat‐free mass; HR, heart rate; ISO, isoproterenol; RMR, resting metabolic rate.

### 2.3 Participants

Written informed consent was obtained from each participant prior to participation in study activities. Sex was self‐reported by participants as male or female at enrollment. Inclusion criteria for both studies included age between 18 and 40 years (inclusive), and absence of overt autonomic, cardiopulmonary, or metabolic disease. Eligibility criteria were created to enroll participants who could safely tolerate systemic β‐AR stimulation.

Specific exclusion criteria for Study 1 included contraindication for use of mirabegron, such as liver and/or kidney dysfunction; use of medications that may unfavorably interact with mirabegron, including thioridazine, flecainide, propafenone, digoxin, and solifenacin succinate; pregnancy or attempting to become pregnant; breastfeeding; habitual use of tobacco/nicotine products (defined as two or more uses within the previous month); bladder dysfunction or medication use related to bladder dysfunction; and sulfite allergy.

Specific exclusion criteria for Study 2 were similar to Study 1, except that there were no restrictions related to mirabegron use or medications that may interact with mirabegron. Participants in Study 2 were also excluded if they had ingested vitamin and/or antioxidant supplements during the previous 4 weeks. Vitamin and antioxidant supplement use was not an exclusion criterion for Study 1 because the vitamin C intervention was specific only to Study 2.

### 2.4 Procedures common to both studies

Both studies utilized a randomized, single‐blind, placebo‐controlled crossover design. To facilitate participant blinding, mirabegron (Study 1) was placed in an opaque gelatin capsule. Vitamin C (Study 2) mixed with saline is colorless and is indistinguishable from saline alone. Following an intake visit, participants reported to the Human Performance Clinical Research Laboratory in the morning shortly after waking on two separate occasions in a randomized order (visit order was randomized by T.R.E. using randomizer.org, accessed in January 2021 for Study 1, and December 2024 for Study 2) for the experimental visits. Prior to the experimental visits, participants fasted for 10 h and abstained from vigorous exercise, caffeine, and alcohol for 22 h. Visits were separated by a minimum of 10 days for Study 1 and 48 h for Study 2. The time between visits was designed to ensure washout of the perturbations (mirabegron half‐life: 50 h (Dehvari et al., 2018), intravenous vitamin C half‐life: approximately 2 h (Lykkesfeldt & Tveden‐Nyborg, 2019)).

### 2.5 Intake visit

Potential research participants provided written informed consent and medical history. Height, mass, and body composition were determined via stadiometer, physician's scale, and dual‐energy X‐ray absorptiometry (DXA; Hologic, Discovery W, QDR Series), respectively. Female research participants completed a pregnancy test prior to body composition assessment. The intake visit concluded with a cardiopulmonary exercise test to volitional fatigue with beat‐by‐beat 12‐lead electrocardiogram (ECG) assessment. A physician reviewed the medical history and ECG to identify possible contraindications for β‐AR stimulation. During the exercise test, indirect calorimetry (Parvo Medics TrueOne 2400) was used for measurement of peak oxygen uptake (VO_2peak_).

### 2.6 Experimental visits

Upon arrival, participants were instrumented for a 3‐lead ECG assessment and blood pressure (BP) measurement (physiological monitor: IntelliVue MP5 Patient Monitor, Philips Healthcare). Participants then rested supine on a hospital bed for 45 min during which resting metabolic rate was measured (indirect calorimetry, ventilated hood technique; Parvo Medics TrueOne 2400), as previously described (Abbotts et al., 2022). Energy expenditure was calculated from oxygen uptake (VO_2_) and carbon dioxide production (VCO_2_) using the Weir Equation (Weir, 1949). The first 15 min were considered a resting phase/habituation; thus, baseline data are reported as the final 30 min of this assessment. Following this baseline measurement, a venous catheter was placed in an antecubital vein for the infusion of isoproterenol (for both studies) and saline, with or without ascorbic acid (Study 2). The perturbation (or placebo) was provided during a lead‐in phase. For Study 1, participants swallowed the β3‐AR agonist and rested for 30 min to allow time for initial agonist absorption. For Study 2, participants rested for 15 min during initiation of the saline or ascorbic acid infusion, which continued through the delivery of isoproterenol. During each isoproterenol delivery, energy expenditure (indirect calorimetry) was continuously measured from minutes 5–30, and heart rate (HR) and BP were recorded at minutes 15 and 30.

Following baseline measurements, and after each isoproterenol dose, the ventilated hood was removed and infrared thermography (FLIR T560‐EST, Teledyne FLIR LLC) was used to capture thermal images of the supraclavicular region. All experiments were undertaken in a temperature‐controlled laboratory (thermostat setting: 22°C).

### 2.7 Infrared thermography analysis

2‐deoxy‐2‐[18F]fluoro‐D‐glucose ([18F]FDG) positron emission tomography/computed tomography (PET/CT) is an established approach for assessing human BAT activity, inferred from glucose uptake (Chen et al., 2016). However, [18F]FDG uptake only considers carbohydrate metabolism, thereby ignoring potential lipid oxidation, and therefore is not truly a direct measure of BAT thermogenesis (i.e., the conversion of chemical energy to heat energy) (Carpentier et al., 2023). PET/CT was also impractical for the present protocol, which required continuous cardiovascular monitoring and intravenous isoproterenol infusion. Therefore, infrared thermography was used to assess supraclavicular skin thermal responses, consistent with an abundance of prior work (Brasil et al., 2020; el Hadi et al., 2016; Haq et al., 2017; Jang et al., 2014; Kwok & Stimson, 2023). The technique involves the use of an infrared camera and specialized software (FLIR Research Studio, version 2026.01.1). Thermographs were taken in duplicate, and the clearer of the two images was used for analysis. As per published methods (Law et al., 2018), the top 10% of hottest pixels for the right and left supraclavicular regions (defined by anatomical position) were combined to determine a mean supraclavicular skin temperature. The thermographs were captured approximately 1 meter from the participant, emissivity was set to 0.98, and the analysis was calibrated using room temperature and relative humidity (Vantage Vue Wireless Weather Station, Davis Instruments). Reference regions of interest (ROIs) were analyzed over the upper sternum and deltoid, and reference region temperatures were calculated as the mean temperature of the entire ROI. The same researcher (T.R.E.) completed all thermographic analysis.

### 2.8 Statistical analysis

Statistical analyses were conducted in R (version 4.5.0) using RStudio (version 2024.12.1 + 563). Participant data from the placebo condition in each study were pooled to provide greater statistical power to evaluate how non‐selective β‐AR stimulation influences thermogenic, cardiovascular, and thermography outcomes. Study (Study 1 vs. Study 2) was included as a covariate in all pooled models to account for potential differences between cohorts. To confirm pooling was appropriate, study interactions with region were tested by likelihood ratio test (study × region and study × region × dose).

Linear mixed models (lme4 (Bates et al., 2015) and lmerTest (Kuznetsova et al., 2017) packages) were used for all primary analyses. For indirect calorimetry and cardiovascular variables, isoproterenol dose was considered as a fixed effect, and participant as a random effect, with both a random intercept and random slope for dose. For infrared thermography, region, dose, and their interaction were considered as fixed effects, with participant as a random intercept. Reference‐region comparisons were restricted to participants with sternum (*n* = 17) and deltoid (*n* = 16) thermographs. Sex differences were evaluated by adding sex and its interaction with dose as fixed effects. The influence of body composition on energy expenditure responses was tested by including body composition variables (% fat, fat mass, fat‐free mass, BMI) and their interaction with dose as fixed effects. The contribution of fat‐free mass to the sex difference was assessed by adding fat‐free mass as a covariate to the sex model. Estimated dose effects are reported as change from baseline; the sex difference in energy expenditure is reported in absolute terms. Significant main effects and interactions were further investigated via Tukey‐adjusted post hoc comparisons (emmeans (Lenth, 2025) package).

Repeated measures correlations (rmcorr (Bakdash & Marusich, 2017) package) were used to assess within‐participant associations between temperature changes, energy expenditure changes, and heart rate changes across ISO doses. These analyses tested whether skin‐temperature changes were associated with whole‐body thermogenesis or a systemic cardiovascular/hemodynamic response.

Intervention effects (Study 1: mirabegron vs. placebo; Study 2: vitamin C vs. placebo) were analyzed within each study using linear mixed models with condition, dose, and their interaction as fixed effects and participant as a random intercept.

To assess whether visually high temperature responses influenced the thermography findings, we performed a sensitivity analysis excluding participants with any regional skin‐temperature increase >2.0°C. ChatGPT (OpenAI; version 5.5) and Claude (Anthropic; versions 4.6–4.8) assisted with R programming and troubleshooting, including mixed models and figure generation. AI‐generated suggestions were reviewed and verified against the original data, analysis outputs, cited sources and author feedback before use. The authors take responsibility for the final content.

Figures were created in R using the ggplot2 package (Wickham, 2016). Energy expenditure is reported in kcal/min (1 kcal = 4.184 kJ). Statistical significance was set at *α* = 0.05.

## 3 RESULTS

### 3.1 Participants and environmental conditions

Data from 19 participants in Study 1 and 9 participants in Study 2 were included in the final analyses (Figure 2). Placebo data from both studies were pooled for analysis of non‐selective β‐AR stimulation (*n* = 28). The regional temperature pattern did not differ between studies (Study × region *p* = 0.22; Study × region × dose *p* = 0.98). Table 1 shows participant characteristics. Experimental visits were conducted under room‐temperature laboratory conditions. Averaged across placebo and intervention visits, laboratory temperature was 22.2°C ± 0.8°C in Study 1 and 22.5°C ± 0.6°C in Study 2, and relative humidity was 43.4% ± 13.1% and 41.7% ± 11.6%, respectively.

**FIGURE 2:**
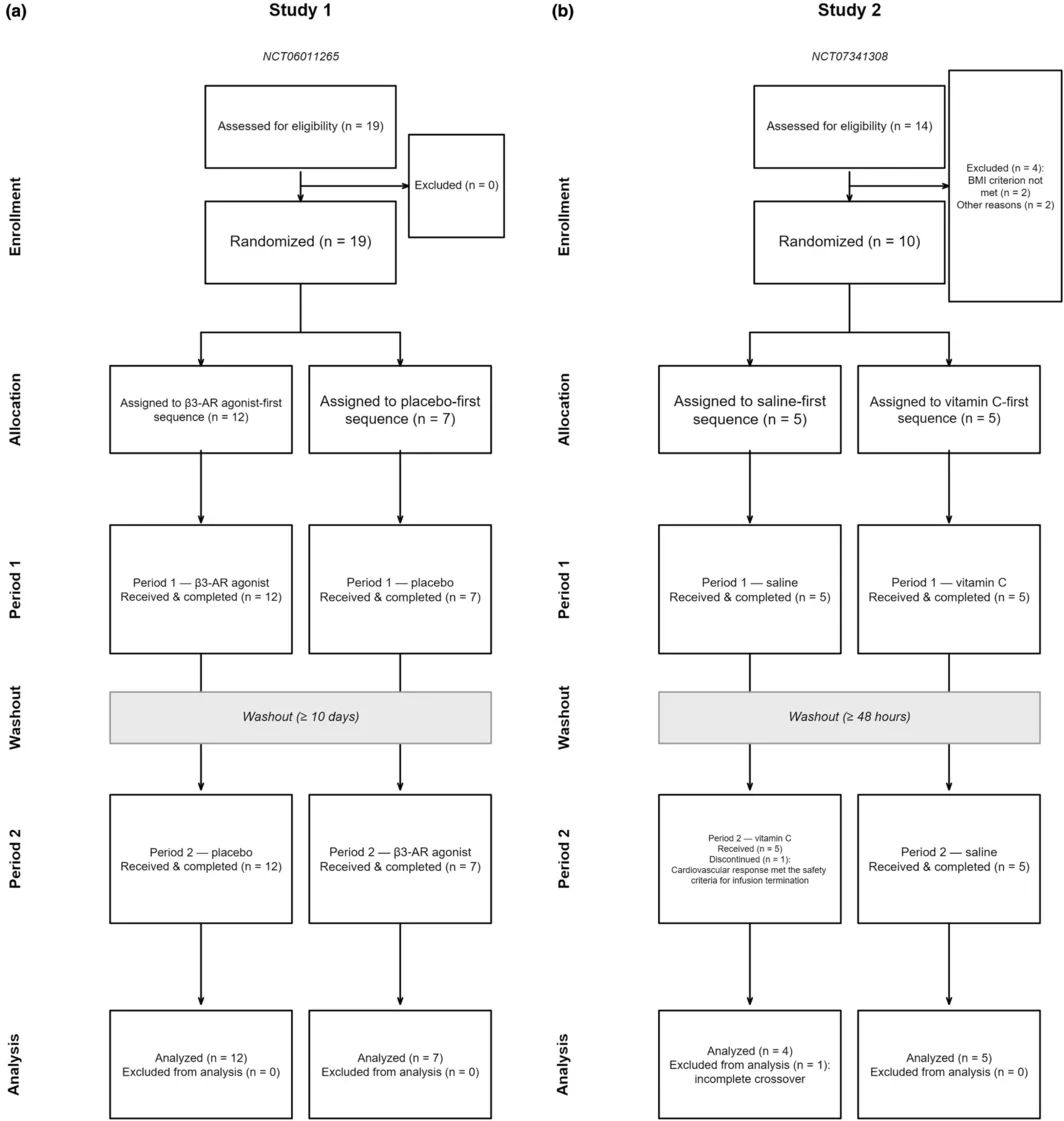
Participant flow diagrams. (a) Study 1 randomized 19 participants; all completed both visits and were analyzed. (b) Study 2 randomized 10 participants; one discontinued due to cardiovascular safety criteria, leaving 9 analyzed. BMI, body mass index; β3‐AR, β3‐adrenergic receptor.

**TABLE 1:** Subject characteristics.

	Combined (*n* = 28)	Study 1 (*n* = 19)	Study 2 (*n* = 9)
Sex (male/female)	14/14	9/10	5/4
Age (years)	26 ± 5	25 ± 4	26 ± 6
Height (m)	1.69 ± 0.10	1.68 ± 0.09	1.70 ± 0.12
Total mass (kg)	72.5 ± 12.4	68.7 ± 11.3	80.5 ± 11.2
Body mass index (kg/m^2^)	25.2 ± 2.7	24.1 ± 2.5	27.7 ± 1.1
Body fat (%)	24.7 ± 5.8	25.0 ± 4.5	24.2 ± 8.2
Fat mass (kg)	17.7 ± 4.0	17.1 ± 3.4	18.9 ± 5.1
Fat‐free mass (kg)	52.6 ± 11.9	49.4 ± 9.9	59.3 ± 13.5
Bone mineral content (kg)	2.3 ± 0.5	2.2 ± 0.4	2.5 ± 0.5
VO_2peak_ (L/min)	2.70 ± 0.88	2.47 ± 0.78	3.19 ± 0.93
VO_2peak_ (mL/kg/min)	46.4 ± 11.4	49.8 ± 11.2	39.2 ± 8.4

*Note*: Values are mean ± SD.

Abbreviation: VO_2_peak, peak oxygen consumption.

### 3.2 Indirect calorimetry

Indirect calorimetry results are shown in Table 2. With the pooled data from both studies, baseline energy expenditure was 1.06 ± 0.26 kcal/min (mean ± SD). As hypothesized, there was an increase in energy expenditure from baseline (ISO 6: 0.09 [0.05, 0.13]; ISO 12: 0.13 [0.09, 0.18]; ISO 24: 0.20 [0.13, 0.26] kcal/min; data are estimated mean differences [95% confidence intervals]; all *p* < 0.001 from baseline). Contrary to our hypothesis, no body‐composition variable (% fat, fat mass, fat‐free mass, BMI) modified the dose‐dependent increase in energy expenditure (dose × body composition interactions: all *p* > 0.17).

**TABLE 2:** Indirect calorimetry responses.

	Pooled	Study 1		Study 2	
	ISO + placebo *n* = 28	ISO + placebo *n* = 19	ISO + mirabegron *n* = 19	ISO + placebo *n* = 9	ISO + vitamin C *n* = 9
EE (kcal/min)					
Baseline	1.06 ± 0.26	1.02 ± 0.26	0.98 ± 0.27	1.13 ± 0.26	1.18 ± 0.24
ISO 6	1.15 ± 0.24	1.11 ± 0.21	1.13 ± 0.23	1.22 ± 0.29	1.25 ± 0.25
ISO 12	1.19 ± 0.24	1.15 ± 0.21	1.18 ± 0.23	1.27 ± 0.30	1.33 ± 0.27
ISO 24	1.25 ± 0.28	1.20 ± 0.23	1.22 ± 0.25	1.35 ± 0.34	1.43 ± 0.29
VO_2_ (mL/min)					
Baseline	221 ± 54	213 ± 53	205 ± 56	236 ± 54	246 ± 50
ISO 6	240 ± 50	233 ± 44	237 ± 49	256 ± 60	263 ± 50
ISO 12	250 ± 52	242 ± 44	249 ± 50	268 ± 64	279 ± 56
ISO 24	264 ± 58	253 ± 49	258 ± 53	286 ± 73	301 ± 60
VCO_2_ (mL/min)					
Baseline	177 ± 44	173 ± 45	163 ± 44	188 ± 41	198 ± 40
ISO 6	191 ± 39	186 ± 34	191 ± 39	201 ± 48	209 ± 45
ISO 12	193 ± 40	188 ± 36	190 ± 36	204 ± 47	216 ± 46
ISO 24	203 ± 44	196 ± 39	197 ± 38	218 ± 53	233 ± 51
RER					
Baseline	0.80 ± 0.04	0.81 ± 0.04	0.80 ± 0.04	0.80 ± 0.03	0.80 ± 0.02
ISO 6	0.80 ± 0.05	0.80 ± 0.06	0.80 ± 0.05	0.78 ± 0.02	0.79 ± 0.04
ISO 12	0.77 ± 0.05	0.78 ± 0.06	0.76 ± 0.05	0.76 ± 0.03	0.77 ± 0.02
ISO 24	0.77 ± 0.04	0.78 ± 0.04	0.77 ± 0.03	0.77 ± 0.03	0.77 ± 0.04
FatOx (g/min)					
Baseline	0.07 ± 0.02	0.07 ± 0.02	0.07 ± 0.03	0.08 ± 0.03	0.08 ± 0.02
ISO 6	0.08 ± 0.03	0.08 ± 0.03	0.08 ± 0.03	0.09 ± 0.02	0.09 ± 0.02
ISO 12	0.09 ± 0.03	0.09 ± 0.03	0.10 ± 0.03	0.11 ± 0.03	0.11 ± 0.02
ISO 24	0.10 ± 0.03	0.09 ± 0.03	0.10 ± 0.03	0.11 ± 0.04	0.11 ± 0.03
CarbOx (g/min)					
Baseline	0.10 ± 0.05	0.10 ± 0.05	0.08 ± 0.04	0.10 ± 0.04	0.11 ± 0.03
ISO 6	0.10 ± 0.06	0.10 ± 0.07	0.11 ± 0.05	0.09 ± 0.04	0.11 ± 0.06
ISO 12	0.08 ± 0.06	0.08 ± 0.07	0.06 ± 0.05	0.07 ± 0.04	0.08 ± 0.04
ISO 24	0.08 ± 0.05	0.08 ± 0.06	0.07 ± 0.03	0.08 ± 0.04	0.09 ± 0.07

*Note*: Values are mean ± SD. In the pooled placebo cohort, energy expenditure increased from baseline by 0.09 kcal/min [95% CI: 0.05, 0.13] at ISO 6, 0.13 kcal/min [0.09, 0.18] at ISO 12, and 0.20 kcal/min [0.13, 0.26] at ISO 24 (all *p* < 0.001). In Study 1, mirabegron increased energy expenditure relative to placebo by 0.07 kcal/min [0.04, 0.10] (main effect of condition, *p* < 0.001), with no condition × dose interaction (*p* = 0.97) These analyses were on changes from baseline values. In Study 2, vitamin C did not alter energy expenditure relative to placebo (0.01 kcal/min [−0.04, 0.05]; main effect of condition, *p* = 0.80). Fat and carbohydrate oxidation assumed negligible protein oxidation.

Abbreviations: CarbOx, carbohydrate oxidation; EE, energy expenditure; FatOx, fat oxidation; ISO, isoproterenol; RER, respiratory exchange ratio; VCO_2_, carbon dioxide production; VO_2_, oxygen consumption.

Males had greater absolute energy expenditure at rest and during non‐selective β‐AR stimulation than females (0.34 kcal/min [0.21, 0.48]; main effect of sex *p* < 0.001), although this difference was no longer significant when fat‐free mass was included as a covariate (estimated sex difference: 0.08 [−0.11, 0.26] kcal/min, *p* = 0.40). Thus, fat‐free mass appeared to explain sex differences in absolute energy expenditure but not interindividual variability in the β‐AR‐mediated thermogenic response.

Additional β3‐AR stimulation (Study 1 only) further augmented the thermogenic response to non‐selective β‐AR stimulation (estimated difference: 0.07 [0.04, 0.10] kcal/min; main effect of condition *p* < 0.001). There was no dose × condition interaction (*p* = 0.97).

Infusion of vitamin C (Study 2) did not augment the thermogenic response to non‐selective β‐AR stimulation (estimated difference: 0.01 [−0.04, 0.05] kcal/min; main effect of condition *p* = 0.80). In absolute terms, energy expenditure differed between visits; however, this difference was already present at baseline and therefore reflected between‐visit variability in resting energy expenditure rather than an effect of the experimental intervention. The pre‐specified change‐from‐baseline analysis showed no augmentation by vitamin C.

### 3.3 Heart rate and blood pressure

Cardiovascular data are presented in Table 3. Participants showed responses typical of non‐selective β‐AR stimulation. Baseline HR was 55 ± 10 beats per minute (bpm) and significantly increased from baseline as dose increased (5 [2, 8]; 11 [7, 15]; and 26 [19, 33] bpm, for ISO 6, 12, and 24; all *p* < 0.01 from baseline). There was no significant dose × sex interaction (*p* = 0.07) nor a main effect of sex on HR (*p* = 0.79).

**TABLE 3:** Cardiovascular responses.

	Pooled	Study 1		Study 2	
	ISO + placebo *n* = 28	ISO + placebo *n* = 19	ISO + mirabegron *n* = 19	ISO + placebo *n* = 9	ISO + vitamin C *n* = 9
HR (bpm)					
Baseline	55 ± 10	57 ± 9	54 ± 9	51 ± 12	53 ± 11
ISO 6	60 ± 9	62 ± 9	61 ± 8	56 ± 9	55 ± 6
ISO 12	67 ± 9	67 ± 9	68 ± 11	65 ± 8	66 ± 6
ISO 24	81 ± 15	82 ± 13	83 ± 14	79 ± 20	83 ± 19
SBP (mmHg)					
Baseline	107 ± 11	108 ± 11	109 ± 11	107 ± 10	112 ± 11
ISO 6	119 ± 12	120 ± 13	117 ± 13	116 ± 10	123 ± 17
ISO 12	125 ± 13	126 ± 14	123 ± 14	123 ± 12	131 ± 16
ISO 24	132 ± 16	133 ± 17	131 ± 15	129 ± 14	136 ± 16
DBP (mmHg)					
Baseline	67 ± 8	68 ± 7	66 ± 7	65 ± 10	65 ± 11
ISO 6	66 ± 8	67 ± 7	65 ± 8	64 ± 10	66 ± 15
ISO 12	65 ± 8	65 ± 6	63 ± 8	65 ± 11	63 ± 13
ISO 24	63 ± 9	62 ± 8	62 ± 8	64 ± 12	64 ± 12

*Note*: Values are mean ± SD. In the pooled placebo cohort, heart rate increased from baseline by 5 bpm [95% CI: 2, 8], 11 bpm [7, 15], and 26 bpm [19, 33] at ISO 6, ISO 12, and ISO 24, respectively (all *p* < 0.01). Systolic blood pressure increased by 11 mmHg [9, 14], 18 mmHg [15, 21], and 24 mmHg [20, 29], respectively (all *p* < 0.001). Diastolic blood pressure decreased at ISO 24 by 4 mmHg [−8, 0] relative to baseline (*p* = 0.04). In Study 1, mirabegron did not modify cardiovascular responses to isoproterenol (condition × dose interactions, all *p* > 0.16). In Study 2, vitamin C did not affect heart rate (*p* = 0.30) or diastolic blood pressure (*p* = 0.52), but increased systolic blood pressure by 5 mmHg [2, 8] relative to placebo (*p* = 0.01).

Abbreviations: DBP, diastolic blood pressure; HR, heart rate; ISO, isoproterenol; SBP, systolic blood pressure.

Baseline systolic blood pressure (SBP) was 107 ± 11 mmHg and increased as dose increased from baseline (11 [9, 14]; 18 [15, 21]; and 24 [20, 29] mmHg; all *p* < 0.001 from baseline). Males had higher systolic blood pressure (17 [10, 24] mmHg, *p* < 0.001), but there was no dose × sex interaction (*p* = 0.07).

Baseline diastolic blood pressure (DBP) was 67 ± 8 mmHg and decreased at ISO 24 compared to baseline (−4 [−8, 0] mmHg; *p* = 0.04). There were no sex differences (*p* = 0.96) nor a dose × sex interaction (*p* = 0.36).

Additional β3‐AR stimulation (Study 1) did not influence these responses (dose × condition interactions: all *p* > 0.16).

Finally, infusion of vitamin C (Study 2) did not change HR or DBP (main effect of condition for HR *p* = 0.30; for DBP *p* = 0.52) but did further increase SBP (estimated difference: 5 [2, 8] mmHg, main effect of condition *p* = 0.01).

### 3.4 Infrared thermography

Infrared thermography data are presented in Figure 3 and Table 4. Baseline skin temperatures were 34.8°C ± 0.7°C in the supraclavicular region, 33.7°C ± 0.7°C at the sternum, and 31.9°C ± 0.9°C at the deltoid. Non‐selective β‐AR agonism increased temperature across every region. There was a significant main effect of dose (*p* < 0.001); post hoc analysis revealed that each increase in dose created an increase in temperature (ISO 12–ISO 6: 0.3°C [0.1, 0.6], *p* = 0.009, ISO 24–ISO 6: 0.7°C [0.4, 0.9], *p* < 0.001, ISO 24–ISO 12: 0.3°C [0.1, 0.6], *p* = 0.006). There was also a significant main effect of region (*p* < 0.001). Post hoc analysis showed that the sternum warmed more than the supraclavicular region (SC) and there was no difference between the deltoid and SC (Sternum–SC: 0.5°C [0.3, 0.8], *p* < 0.001; Deltoid–SC: 0.2°C [0.0, 0.5], *p* = 0.11; Sternum–Deltoid: 0.3°C [0.0, 0.6], *p* = 0.02) on skin temperature change from baseline. Excluding visually high responders did not alter the regional thermography findings. Sternum warming remained greater than supraclavicular warming after removal of these participants, indicating that the conclusion was not driven by visually high‐temperature observations.

**FIGURE 3:**
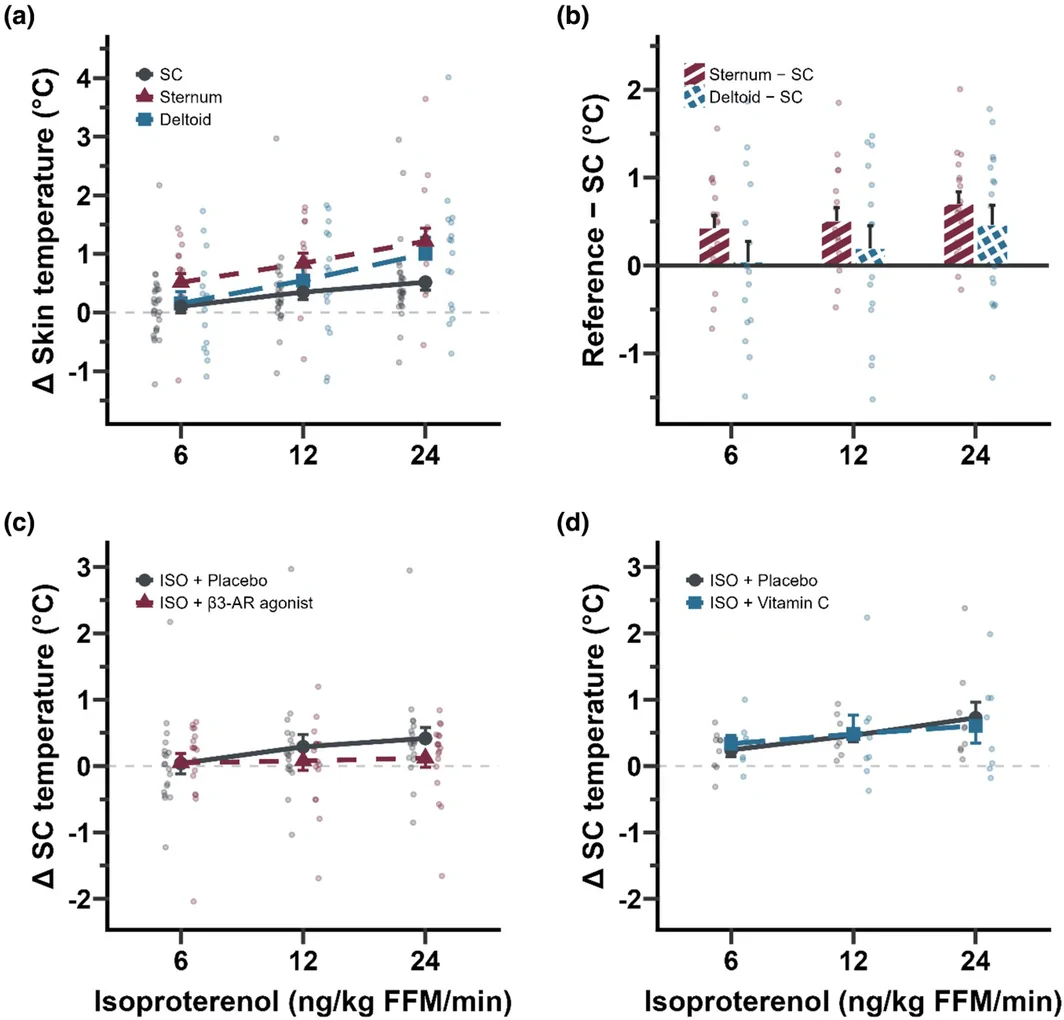
Infrared thermography responses during β‐adrenergic stimulation. Skin‐temperature changes from baseline are shown during pooled placebo isoproterenol infusion (a), as reference‐region minus supraclavicular differences (b), during Study 1 placebo versus β3‐adrenergic receptor agonist treatment (c), and during Study 2 placebo versus vitamin C treatment (d). Points represent individual observations; group values are mean ± SEM. In the pooled placebo analysis, there were main effects of isoproterenol dose (*p* < 0.001) and region (*p* < 0.001), with no dose × region interaction (*p* = 0.47). Across doses, sternum warming exceeded supraclavicular warming by 0.5°C [95% CI: 0.3, 0.8] (*p* < 0.001), whereas deltoid and supraclavicular warming did not differ (0.2°C [0.0, 0.5], *p* = 0.11). In Study 1, additional β3‐AR stimulation did not increase supraclavicular temperature relative to placebo (0.1°C [−0.1, 0.40], *p* = 0.29). In Study 2, vitamin C had no effect on supraclavicular temperature (main effect of condition, *p* = 0.29). FFM, fat‐free mass; ISO, isoproterenol; SC, supraclavicular; β3‐AR, β3‐adrenergic receptor.

**TABLE 4:** Regional skin temperature responses.

	Baseline	ISO 6	ISO 12	ISO 24
Supraclavicular	34.8 ± 0.7	34.9 ± 0.4	35.1 ± 0.4	35.3 ± 0.6
Sternum	33.7 ± 0.7	34.2 ± 0.6	34.6 ± 0.7	34.9 ± 1.0
Deltoid	31.9 ± 0.9	32.0 ± 1.2	32.4 ± 1.3	32.9 ± 1.3

*Note*: Values are mean ± SD in°C from the pooled placebo condition. Statistical analyses were performed on change from baseline. Skin temperature increased with isoproterenol dose (main effect of dose, *p* < 0.001) and differed among regions (main effect of region, *p* < 0.001). Across doses, sternum warming exceeded supraclavicular warming by 0.53°C [95% CI: 0.27, 0.79] (*p* < 0.001). Sample sizes varied by region and dose because reference‐region analysis was retrospective: supraclavicular *n* = 27–28, sternum *n* = 16–17, and deltoid *n* = 15–16.

Abbreviation: ISO, isoproterenol.

Additional β3‐AR stimulation (Study 1) did not increase SC temperature relative to placebo (0.1°C [−0.1, 0.4], *p* = 0.29). However, when comparing thermography regions (main effect of region *p =* 0.006), consistent with the pooled analysis, the sternum warmed more than SC (0.4°C [0.1, 0.7], *p* = 0.02) and there was no difference between SC and deltoid temperatures (*p =* 0.94).

Finally, infusion of vitamin C (Study 2) had no effect on temperatures (main effect of condition *p* = 0.29). However, non‐selective β‐AR stimulation led to a significant increase in regional temperatures (main effect of dose *p* < 0.001), with greater warming in the sternum than in SC (0.6°C [0.3, 0.9], main effect of region *p* < 0.001) and no difference between SC temperature and deltoid temperature (0.1°C [−0.3, 0.4], *p =* 0.87).

There was no main effect of sex on temperature in the non‐selective β‐AR stimulation cohort, the additional β3‐AR stimulation group, or the vitamin C group (*p* = 0.29, 0.85, and 0.39, respectively).

### 3.5 Thermography associations with energy expenditure and heart rate

Repeated measures correlations were used to assess the within‐participant relationship between temperature changes and energy expenditure changes across the three thermography regions (Figure 4). Temperature changes were positively associated with energy expenditure changes in all regions (SC: *r* = 0.50 [0.27, 0.67]; Sternum: *r* = 0.59 [0.31, 0.77]; Deltoid: *r* = 0.60 [0.32, 0.79]; all *p* < 0.001). Temperature changes in all three regions were also associated with changes in HR (SC: *r* = 0.76 [0.62, 0.85]; Sternum: *r* = 0.82 [0.66, 0.91]; Deltoid: *r* = 0.76 [0.57, 0.88]; all *p* < 0.001).

**FIGURE 4:**
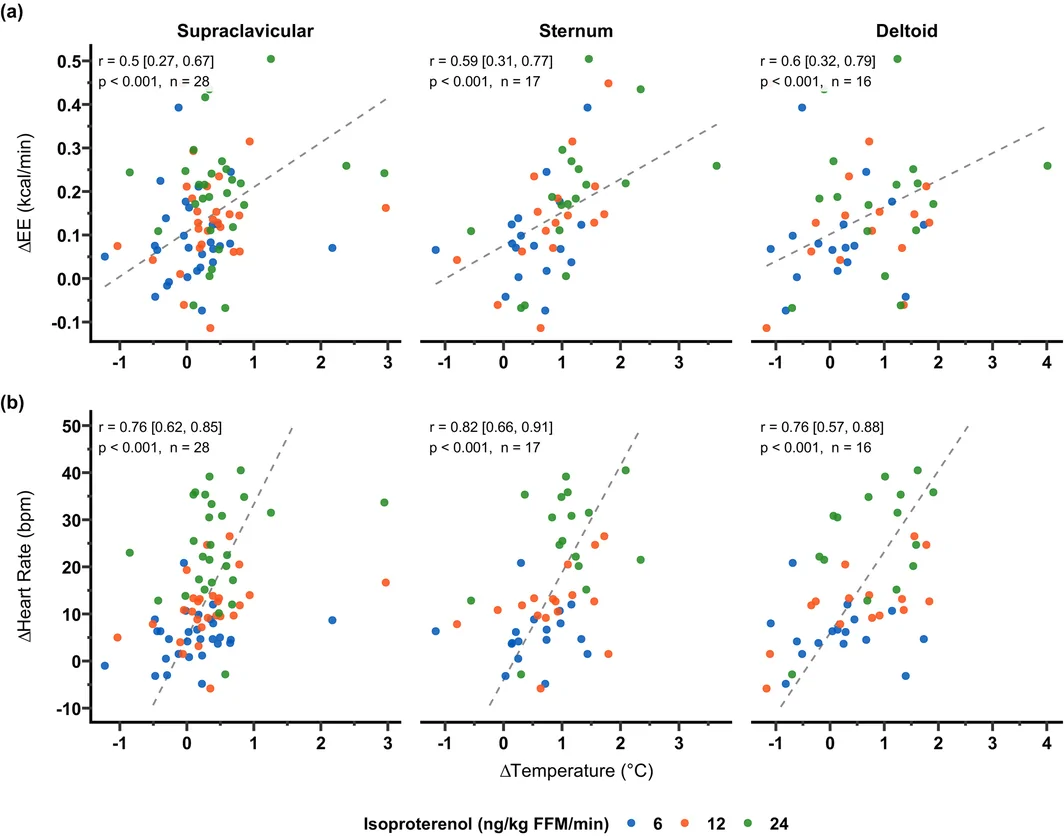
Associations between regional skin temperature, energy expenditure, and heart rate. Repeated‐measures correlations assessed associations between regional skin temperature and energy expenditure (a) or heart rate (b). Points represent participant‐dose observations; dashed lines represent repeated‐measures correlation slopes. EE, energy expenditure; FFM, fat‐free mass; HR, heart rate; ISO, isoproterenol.

## 4 DISCUSSION

The findings of these studies were (1) non‐selective β‐AR stimulation increased energy expenditure. (2) Body composition did not modify the thermogenic response to non‐selective β‐AR stimulation. (3) Males had greater absolute energy expenditure than females across resting and β‐AR‐stimulated conditions; this difference was no longer significant after adjustment for FFM. (4) Non‐selective β‐AR stimulation increased supraclavicular skin temperature, but this warming was not preferential, as reference regions without known BAT depots warmed to a similar or greater extent. (5) Regional temperature changes were more strongly associated with heart rate than energy expenditure, consistent with a shared cardiovascular or hemodynamic influence rather than region‐specific metabolism; and (6) additional β3‐AR stimulation, but not intravenous vitamin C, augmented the thermogenic response to non‐selective β‐AR stimulation. Together, these findings indicate that non‐selective β‐AR stimulation produced the expected systemic physiological response, but supraclavicular skin thermography did not isolate a BAT‐specific thermal signal.

Participants exhibited responses typical for young, healthy adults undergoing non‐selective β‐AR stimulation (Stob, Bell, et al., 2007). Energy expenditure (9%, 12%, and 18%), HR (9%, 22%, and 47%), and SBP (11%, 17%, and 23%) all increased above resting baseline values at the three escalating doses. Respiratory exchange ratio, a crude indicator of substrate oxidation, declined from 0.80 to 0.77, suggestive of β‐AR‐mediated increases in fat oxidation (Blaak et al., 1993; Schiffelers et al., 2001). Finally, DBP decreased by 6% (4 mmHg) at our highest dose, which is perhaps suggestive of β2‐AR‐mediated vasodilation (Limberg et al., 2022). These responses are consistent with previous investigations that used graded non‐selective β‐AR stimulation in healthy adults (Newsom et al., 2010; Stob, Seals, et al., 2007).

No measured component of body composition explained the β‐AR‐mediated increase in energy expenditure. This is contrary to our hypothesis that was based on prior evidence that BAT prevalence and adrenergic responsiveness decline with greater adiposity (Blaak et al., 1995; Cypess et al., 2009). However, despite a wide range of BMI values (20–30 kg/m^2^), total body fat percentage did not vary considerably (25% ± 6%), and it is possible that the range was too narrow to determine a meaningful correlation, or alternatively, no association exists in this population.

Although adiposity did not explain the thermogenic response, sex was associated with energy expenditure. Despite the β‐AR stimulation being normalized to FFM, males had greater energy expenditure than females. This sex difference disappeared when FFM was included as a covariate, which was itself a significant predictor of energy expenditure (*β* = 0.022 kcal/min per kg FFM, *p* < 0.001). A potential explanation for the apparent overbearing influence of FFM may relate to the linear manner by which isoproterenol was scaled to FFM. Future studies may wish to consider an allometric approach to isoproterenol dosing.

After confirming the expected systemic response to non‐selective β‐AR stimulation, the central finding was that supraclavicular skin temperature lacked regional specificity during β‐AR stimulation. The premise behind using infrared thermography to infer BAT activity is that the supraclavicular depot has the greatest abundance of BAT in humans (Cypess et al., 2009). Because BAT is thermogenic, increased BAT metabolic activity may contribute to warming of the overlying supraclavicular skin. Critically, for supraclavicular thermography to be a surrogate measure for BAT activation, the supraclavicular space should warm more than tissues without appreciable BAT. These “reference regions” serve as a control comparison. The gradient between the supraclavicular region and the reference regions should become larger as BAT is further activated. However, the use of reference regions has been inconsistent (Jimenez‐Pavon et al., 2019). Some studies report only the supraclavicular signal (Ang et al., 2017; da Rosa et al., 2023; Haq et al., 2017; Hartwig et al., 2017; Malpique et al., 2019; Robinson et al., 2014) or report the supraclavicular signal and a reference region, but do not reconcile the two to provide an estimate of BAT activity (Scotney et al., 2017). Other studies in this area, if they include a reference region, use either the deltoid or sternum, under the assumption they are BAT‐free (Leitner et al., 2017). We compared supraclavicular skin temperature with both a bony (sternum) and a muscular (deltoid) reference region during graded β‐AR stimulation. In our study, the supraclavicular region did not warm to a greater extent compared to the reference regions. Supraclavicular skin temperature increased by 0.56°C at peak stimulation, but the sternum and deltoid warmed 0.70°C and 0.47°C more than the supraclavicular region, respectively. For infrared thermography to quantify β‐AR‐mediated BAT activation at room temperature, the BAT signal should have been greater than the reference region. This was not the case with our investigation.

There are several plausible explanations for our findings. First, non‐selective β‐AR stimulation produces β‐AR‐mediated vasodilation in skeletal muscle, increasing cutaneous and limb blood flow (Harvey et al., 2014). Combined with a rise in cardiac output (implied by the increase in HR), this could raise skin blood flow and warm the overlying skin. Alternatively, β‐AR stimulation increases muscle metabolism (Blaak et al., 1994). This may have caused an increase in the deltoid skin temperature concomitant with potential BAT activation in the supraclavicular region, making the two regions indistinguishable. β‐AR stimulation of cardiac tissue, alongside muscle metabolism of the pectoral muscles and increased blood flow combined, may explain why the skin temperature adjacent to the sternum warmed the most. A different explanation may relate to supraclavicular adipose serving as a thermal insulator. Deep BAT depots may not be superficial enough for thermogenesis to reach the skin (Gatidis et al., 2016). It is possible that BAT was fully activated, but confounding physiological influences make infrared thermography the wrong tool to detect it under these experimental conditions.

The correlation analyses could be interpreted to suggest that the thermal response was more vascular/systemic than BAT‐specific. In our study, temperature changes were moderately correlated with changes in energy expenditure (SC *r* = 0.50 [0.27, 0.67]; sternum *r* = 0.59 [0.31, 0.77]; deltoid *r* = 0.60 [0.32, 0.79]; all *p* < 0.001) but strongly correlated with heart rate (SC *r* = 0.76 [0.62, 0.85]; sternum *r* = 0.82 [0.66, 0.91]; deltoid *r* = 0.76 [0.57, 0.88]; all *p* < 0.001). Notably, the supraclavicular region did not show a stronger association with energy expenditure than either reference region, despite being the expected site of BAT activity. This pattern is more consistent with a shared vascular driver, such as increased cardiac output and cutaneous blood flow, rather than region‐specific metabolic activity.

The intervention data further demonstrate that changes in energy expenditure and supraclavicular skin temperature can dissociate. Additional β3‐AR agonism (Study 1) did augment energy expenditure (an additional 0.07 kcal/min, *p* < 0.001). Yet there was no difference in supraclavicular skin temperature between conditions (*p* = 0.29), nor any difference in cardiovascular responses (all *p* > 0.16). β3‐AR signaling is a primary driver of BAT activation in rodents, although the receptor subtype responsible for BAT activation in humans is controversial (Cero et al., 2021). In any case, energy expenditure increased without an increase in supraclavicular skin temperature. This dissociation could lead to two possible interpretations: the increase in energy expenditure was mediated by non‐BAT tissues or BAT was indeed activated but infrared thermography was unable to isolate it. The supraclavicular region is not thought to be the only place where BAT exists, so it is plausible that other BAT‐containing regions were activated to help drive increased energy expenditure. In either case, supraclavicular skin thermography was unable to distinguish between these possibilities, highlighting its limitations as a tool for inferring changes in energy expenditure to reflect BAT activation at normal room temperature.

Vitamin C (Study 2) did not augment the thermogenic response to non‐selective β‐AR stimulation. In previous work, intravenous ascorbic acid, administered during isoproterenol infusion, augmented β‐AR‐mediated thermogenesis in sedentary adults such that it was no longer lower than thermogenesis in recreationally active adults (Bell et al., 2006). This enhancing effect was observed in an older, sedentary cohort. Our participants, though of similar BMI, were younger and had a greater VO_2peak_, indicative of superior aerobic conditioning (Age: 26 vs. 51 years; VO_2peak_: 39 vs. 29 mL/kg/min in the sedentary group from that study (Bell et al., 2006)). The lack of effect of intravenous vitamin C may reflect lower basal oxidative stress in our young, healthy participants. Prior studies showing that vitamin C augments β‐AR‐mediated thermogenic (Bell et al., 2006) or cardiac (Mak & Newton, 2001; Shinke et al., 2007) responses imply that this effect may be context dependent.

This study has several limitations. An independent imaging‐based measure of BAT activity was not included. [18F]FDG PET/CT would have allowed us to compare infrared thermography responses with an established imaging‐based marker of BAT activity during non‐selective β‐AR stimulation. Cutaneous blood flow and muscle metabolism were not measured directly, so the proposed vascular and muscular contributions, while physiologically plausible, remain inferred from previous studies using non‐selective β‐AR stimulation. Additionally, reference region analysis was retrospective, and the analyzable subset was limited by image availability; the supraclavicular region was analyzed in all participants (*n* = 28), whereas the sternum (*n* = 17) and deltoid (*n* = 16) were available in smaller subsets. All thermographic analyses were completed by the same investigator, so there is a risk of bias. However, most of the analysis was algorithmic, and expectancy bias would be expected to skew results toward the infrared thermography data supporting our initial hypotheses, which was not the case in our investigation. Study 1 did not include a mirabegron‐only condition, and participants were studied before mirabegron may have reached peak concentration; therefore, we cannot determine whether later β3‐AR effects would have emerged. Antioxidant status was not assessed in Study 1. This may mean that our results are confounded if there were differences in oxidative stress in the same participant between conditions. Brown adipose tissue activity can increase with acclimatization. It is possible that our pooled data may reflect the influence of seasonality, although this does not seem likely to mediate our findings related to the failure of supraclavicular infrared thermography to detect BAT activation during non‐selective β‐AR stimulation at room temperature. Participant skin temperatures may fluctuate with time or if they became drowsy during the experiment. For safety reasons, the infusions of isoproterenol always started with the lowest dose (6 ng/kg ffm/min); thus, we cannot disentangle the effects of the dose increasing from time elapsing. Finally, these findings apply to non‐selective pharmacological stimulation at room temperature. BAT has been studied with thermography primarily under cold exposure, which promotes cutaneous vasoconstriction and may improve regional specificity (Charkoudian, 2003). Despite these limitations, the central observation remains that supraclavicular skin warming was not preferential relative to anatomically distinct control regions during non‐selective β‐AR pharmacological stimulation.

## 5 CONCLUSIONS

To conclude, non‐selective β‐AR stimulation increased thermogenesis but infrared thermography was unable to distinguish BAT‐specific thermogenesis from broader regional warming. BAT may have been activated, but infrared thermography was unable to isolate that signal. Therefore, supraclavicular skin warming alone should be interpreted cautiously when used as evidence of BAT activation. Future studies using infrared thermography should evaluate supraclavicular skin warming relative to non‐BAT reference regions rather than in isolation. As interest in BAT as a metabolic target continues to grow, the methods used to assess it must be able to distinguish BAT‐specific signals from systemic ones.

## AUTHOR CONTRIBUTIONS

**Taylor R. Ewell:** Conceptualization; data curation; formal analysis; investigation; methodology; project administration; validation; visualization. **Christina M. N. Cheng:** Data curation; formal analysis; investigation; validation; visualization. **Matthew C. Bomar:** Investigation. **Allison M. Tripure:** Investigation; visualization. **David M. Brown:** Formal analysis; investigation. **Reagan L. Brown:** Formal analysis; investigation. **David P. Thomson:** Investigation; resources; supervision. **Blair D. Johnson:** Conceptualization; funding acquisition; methodology; resources. **Christopher Bell:** Conceptualization; funding acquisition; investigation; methodology; project administration; resources; supervision.

## FUNDING INFORMATION

This work was supported by the Office of Naval Research (N00014‐21‐1‐2276).

## CONFLICT OF INTEREST STATEMENT

The authors declare no conflicts of interest. Dr. Johnson is an Associate Editor at Physiological Reports and was blinded from reviewing or making decisions for the manuscript. Another Editor oversaw the manuscript process for this article.

## ETHICS STATEMENT

The Colorado State University Institutional Review Board approved both protocols (Study 1: #2981, approved January 2021; Study 2: #6115, approved December 2024).

## CONSENT

All participants provided written informed consent prior to participation.

## ARTIFICIAL INTELLIGENCE

TRE used ChatGPT (OpenAI; Version 5.5) and Claude (Anthropic; Opus 4.6–4.8) to suggest wording revisions and to draft and troubleshoot R code. AI‐generated suggestions were reviewed and verified against the original data, analysis outputs, cited sources, and author feedback before use. The authors take responsibility for the final content.

## Data Availability

The data that support the findings of this study are available from the corresponding author upon reasonable request.
